# Multiple Melanomas on Speckled Lentiginous Nevus: A Systematic Review and a Case Report

**DOI:** 10.3390/jcm14186366

**Published:** 2025-09-09

**Authors:** Simona Frațilă, Ovidiu Țica, Ioana Adela Rațiu, Alexandra Ardelean

**Affiliations:** 1 Faculty of Medicine and Pharmacy, University of Oradea, 1st December Square 10, 410073 Oradea, Romania; ioana.ratiu@didactic.uoradea.ro; 2 Clinic of Dermatology, Emergency County Clinical Hospital of Bihor, 37 Republicii Street, 410167 Oradea, Romania; ardelean.alexandra@student.uoradea.ro; 3 Department of Pathology, Emergency County Clinical Hospital of Bihor, 410169 Oradea, Romania; 4 Nephrology Department, Emergency County Clinical Hospital of Bihor, 12 Corneliu Coposu Street, 410469 Oradea, Romania; 5 Doctoral School of Biomedical Sciences, University of Oradea, 410087 Oradea, Romania

**Keywords:** melanoma, multiple melanoma, speckled lentiginous nevus, nevus spilus, multiple melanomas, systematic review, congenital nevus

## Abstract

**Background:** Speckled lentiginous nevus (SLN), also known as nevus spilus (NS), is a variant of congenital melanocytic nevus. Although historically considered to have low malignant potential, recent studies have reported melanoma arising within SLN. This study presents a systematic review of multiple melanomas occurring in association with SLN and includes a representative clinical case. **Methods:** We conducted a systematic review in accordance with the Preferred Reporting Items for Systematic Reviews and Meta-Analyses (PRISMA) guidelines. A comprehensive search of PubMed, Scopus, Web of Science, and Google Scholar was performed from 1957 to 2025 using the terms “melanoma” and “nevus spilus” or “speckled lentiginous nevus.” Filters were applied for original reports, case series, and case reports. Studies were selected based on predefined criteria, with data independently extracted by two reviewers. A case of a 66-year-old male with three melanomas (two within and one outside SLN) over nine years is also presented. Because the evidence base consisted almost exclusively of case reports and small series, meta-analysis and formal risk-of-bias assessment were not feasible; findings were therefore synthesized qualitatively. **Results:** We first describe an illustrative case of a 66-year-old male who developed three melanomas (two within and one outside SLN) over a nine-year period, underscoring the challenges of diagnosis and long-term monitoring. In the systematic review, we identified 41 eligible publications describing 51 patients, and in our illustrative case, we identified a total of 52 with melanoma on SLN; 9/52 (17.3%) developed multiple melanomas (24 total), and in our illustrative case, we identified a total of 52. Most were male (seven of nine), with the first melanoma diagnosed at a mean age of 52.4 years. The majority (21/24) occurred within SLNs ≥5 cm and were of the superficial spreading type (16/17 where specified). Of 24 tumors, 19 (79.2%) were synchronous, and among the 16 invasive melanomas, the mean Breslow thickness was 1.17 mm (median 0.95 mm, IQR 0.56–1.40 mm). **Conclusions:** Large or segmental SLNs may carry a clinically relevant risk for developing multiple melanomas. Regular full-body skin examinations and dermoscopic monitoring are recommended for early detection and management. As the synthesis is based mainly on case reports and small series, these conclusions are necessarily descriptive and exploratory, providing a qualitative mapping of the available evidence rather than definitive risk estimates.

## 1. Background

Speckled lentiginous nevus (SLN) is a relatively common variant of congenital melanocytic nevus, also known as nevus spilus (NS) [1,2,3]. It is present at birth or early infancy as a uniform light brown macule or patch that develops, over the next months or years of life, superimposed, scattered darkly pigmented macules and papules [4,5]. According to the clinical appearance of SLN, two major types have been described: macular and papular [6]. SLN has a prevalence that varies with age, similar to congenital nevi, from 0.2% in newborns to 2.3% in adults [2,7,8], and they are classified as small (<1.5 cm), medium (1.5–19.9), large (>20 cm), and segmental or zosteriform (corresponding to a dermatome) [9]. SLN has a predilection for the trunk and extremities, but also the face or oral mucosa may be involved [10,11,12].

Speckled lentiginous nevus (SLN) remains a complex condition with a natural history that is not yet fully understood [9]. SLN is a dynamic pigmented lesion [13] that can evolve over time due to factors such as aging, sun exposure, or hormonal changes like pregnancy [9]. This variability has led to comparisons with a ‘melanocytic garden,’ where various melanocytic lesions, including melanoma, may arise [9].

The risk for melanoma developing in SLN was still considered close to zero (0.13–0.2%) 30–40 years after Perkinson N. described the first melanoma on SLN in 1957 [14,15,16]. Until now, about 40 cases of melanoma arising on the SLN surface have been described in the literature, and increased awareness of these lesions as precursors of melanoma [16,17,18,19,20,21,22]. In some cases, the outcome was fatal [19,20,23]. These publications are case reports or small case series of single or multiple melanomas on SLN that also include small reviews of published cases [20,22,24,25,26,27,28,29,30,31]. There is no updated review in the literature to sum all the cases of melanoma on SLN, and no review about multiple melanomas developing in SLN has been published yet. This systematic review aims to identify and synthesize all published cases of multiple melanomas arising in association with speckled lentiginous nevus (SLN) to assess risk patterns, patient demographics, lesion characteristics, and clinical outcomes. In addition to the literature synthesis, we also present an illustrative clinical case that highlights the diagnostic and management challenges of melanoma within SLN. This case is presented first in the Results section, providing clinical context before transitioning into the systematic review findings.

## 2. Methods

We conducted a systematic review following the Preferred Reporting Items for Systematic Reviews and Meta-Analyses (PRISMA) guidelines [32], supplemented by a representative case. A comprehensive literature search was carried out across four major databases (PubMed, Scopus, Web of Science, and Google Scholar), spanning publications from 1957 to 2025. This review was conducted in accordance with the Preferred Reporting Items for Systematic Reviews and Meta-Analyses (PRISMA) 2020 statement (Page et al., 2021 [32]).

### Information Sources and Search Strategy

We systematically searched PubMed/MEDLINE, Scopus, Web of Science Core Collection, and Google Scholar for reports published between 1 January 1957 and 28 February 2025. The last search was performed on 28 February 2025. Searches were restricted to articles published in English, French, German, or Spanish.

The PubMed/MEDLINE search strategy was: ((“Melanoma” [Mesh] OR melanoma [tiab]) AND (“nevus spilus” [tiab] OR “naevus spilus” [tiab] OR “speckled lentiginous nevus” [tiab] OR “speckled lentiginous naevus” [tiab])) AND (Case Reports [Publication Type] OR “case report” [tiab] OR “case series” [tiab]) AND (“1957/01/01” [Date-Publication]: “2025/02/28” [Date-Publication]).

For Scopus, Web of Science, and Google Scholar, we used the keywords: melanoma AND (nevus spilus OR naevus spilus OR speckled lentiginous nevus OR speckled lentiginous naevus), with results limited to the same date range and languages. In Google Scholar, the first 200 results were screened by relevance, followed by an additional pass sorted by date.

After removal of duplicates, titles and abstracts were screened independently by two reviewers, with adjudication by a third in case of disagreement. Full texts were retrieved for potentially eligible articles. Inclusion criteria were original case reports or case series describing melanoma arising within SLN. Exclusion criteria were review articles, letters duplicating previously published cases, animal studies, melanomas arising in other types of nevi, and records without accessible full text.

Predefined inclusion and exclusion criteria were applied, and data extraction was conducted independently by two reviewers. The findings are synthesized to provide an evidence-based characterization of multiple melanomas arising within SLN. Given the rarity and clinical heterogeneity of the included cases (primarily case reports and small series), this review adopts a qualitative synthesis approach within a systematic framework. A formal meta-analysis was not feasible due to data limitations. This review was not registered in PROSPERO, given its retrospective, case-report-driven nature and the rarity of the condition. Future updates may consider registration for enhanced transparency. Given the nature of the included studies (case reports and series), a formal risk of bias assessment was not feasible; however, data completeness and clinical detail were considered during selection.

After clinical and dermoscopic examination (Dermlite3 and Dermlite5, 3Gen, Inc., San Juan Capistrano, CA, USA), the lesions were photographed with the mobile phone’s camera and then excised in our clinic. The excised sample was prepared for histopathological examination by standard fixation of human tissue, vacuum histoprocessing, automated staining (Hematoxylin–Eosin) with coverslipping, and automated immunohistochemistry (Leica Bond Max platforms with Novocastra antibodies). The Leica DM 3000 LED microscope (Leica Microsystems, Wetzlar, Germany) was used for the microscopic examination, the Leica camera for the histopathological photos, and the camera software was used to measure the Breslow thickness. The patient signed and agreed to the participation in scientific reports according to the national and legal requirements. The study was conducted in accordance with the Declaration of Helsinki and approved by the Institutional Ethics Committee of SCJU BH (protocol number 25630; date of approval 22 August 2024).

The systematic review concerning multiple melanomas that develop within SLN was performed according to the Preferred Reporting Items for Systematic Reviews and Meta-Analyses (PRISMA) guidelines [32]. A comprehensive search was performed between August 2024 and February 2025. For each study, two investigators (S.F. and A.A.) independently extracted and tabulated data using a standardized data extraction form. Title and abstract screening, as well as full-text eligibility assessment, were conducted independently by the same reviewers. Any discrepancies or missing data were resolved through discussion and, when necessary, adjudicated by a third reviewer (O.T.) regarding the original publication. The reports were searched by using the following keywords: melanoma and “nevus spilus” or “speckled lentiginous nevus” on PubMed, Scopus, Web of Science, and Google Scholar. To retrieve the most relevant original articles, filters were applied for case reports and case series (melanomas, “case reports”, “case series”). Further on, irrelevant articles (out of scope, unavailable abstract) were excluded manually in each database by reading the title and the abstract. Records without an accessible full text were excluded at the eligibility stage. New studies were identified manually through citation search, and from these, the titles with unavailable full text were excluded. The eligible records were original reports or case series of melanoma arising on SLN (nevus spilus), published in peer-reviewed journals, from 1957 (when the first case was reported—Perkinson) to 2025 (Nocivin), in English, French, German, and Spanish. From all the eligible retrieved records, we selected those articles that presented more than one melanoma arising on a speckled lentiginous nevus. The following data were extracted by A.A and then reviewed by S.F. for accuracy: patients’ demographic data: gender, age when SLN was detected and age(s) when each melanoma was diagnosed; for speckled lentiginous nevus (nevus spilus): size, topography, and type; for melanomas: number of melanomas/patient, the site of development—within SLN or outside, histological type, Breslow index, and evolution. Exclusion criteria: articles with unavailable abstracts or full content; reports of melanoma arising in other types of melanocytic nevi; cases of SLN that developed single melanoma; review articles or letters to the editor including already published original cases; and studies involving animal subjects.

Cases of NS-like CMN (nevus spilus-like congenital melanocytic nevus) were excluded because they represent a distinct clinical and genetic entity, associated with larger congenital melanocytic nevi and HRAS mutations, and carry a different melanoma risk profile compared with classic SLN. 

This study is a systematic review of case reports and small case series, conducted in accordance with PRISMA guidelines. Given the rarity of multiple melanomas arising within speckled lentiginous nevus and the reliance on individual case reports, a quantitative meta-analysis and formal risk of bias assessment were not feasible. Instead, we performed a qualitative synthesis of the available evidence, focusing on mapping clinical patterns, lesion characteristics, and outcomes.

## 3. Results

### 3.1. Illustrative Case Report

**Figure 1:**
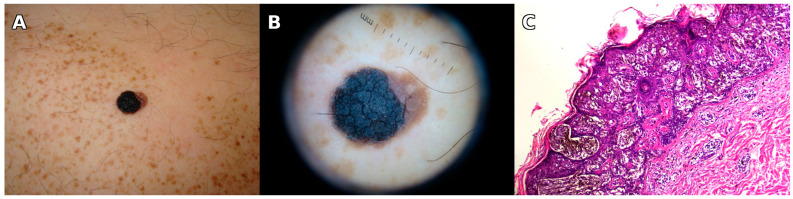
**First melanoma (2013) within the SLN** = Superficial spreading melanoma on the left shoulder within SLN in a 66-year-old man. (**A**) High-resolution clinical image showing the entire speckled lentiginous nevus on the left shoulder, with a close-up focus on the evolving melanoma lesion (10×8 mm) demonstrating eccentric black pigmentation against the SLN background. (**B**) Dermoscopic image showing an irregular, structureless lesion with an eccentric black area and peripheral asymmetry, suggestive of melanoma. (**C**) Histopathology (H&E stain) reveals confluent atypical melanocytes with pagetoid spread and invasion into the upper dermis, consistent with superficial spreading melanoma.

We report on the case of a single patient who developed three separate melanomas over 9 years. The first lesion appeared in 2013 within a speckled lentiginous nevus (SLN), followed by two additional melanomas in 2022 (one within the SLN, one on the scalp outside the nevus). This case illustrates the natural history, diagnostic challenges, and importance of long-term follow-up in patients with SLN. His SLN appeared in early childhood, and eight years before this presentation, a pigmented lesion was removed from this area. The histopathological diagnosis was compound pigmented nevus. A dermatological examination in October 2013 revealed an SLN extending from the posterior midline to the proximal half of the left arm and a few darker spots. The one indicated by the patient as changing, located on the left shoulder, was a black, round, flattened plaque, 10 × 8 mm, with a verrucous surface, showing on the lateral margin a yellowish-brown crescent and a light brown patch. Dermoscopic examination revealed an irregular, structureless lesion with an eccentric black area (Figure 1). The first melanoma was identified in 2013 on the left shoulder within the SLN (Figure 1). A higher-resolution clinical image was obtained to better illustrate the full extent of the SLN and the onset of melanoma within it (Figure 1A).

It was clinically diagnosed as a seborrheic keratosis and excised to exclude malignancy, due to the recent change. The histopathological report revealed an asymmetrical melanocytic proliferation with large nests of epithelioid melanocytes, atypical mitosis, and pagetoid spread. No epidermal ulceration or dermal invasion was spotted. The histological diagnosis was in situ melanoma with a superficial spreading pattern. A wide re-excision was carried out, and no remaining tumor was identified.

**Figure 2:**
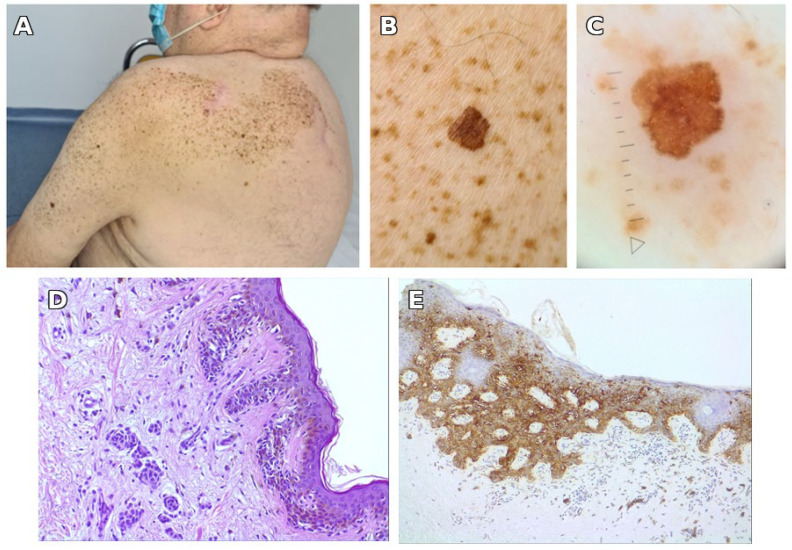
**Second melanoma (2022) within the SLN.** Second superficial spreading melanoma in the same patient with a speckled lentiginous nevus, after 9 years. (**A**) Overview showing an extensive speckled lentiginous nevus on the shoulder and upper arm. A few darker pigmented spots are present; the enlarging one on the lower posterior arm raised suspicion. (**B**) Clinical close-up of the evolving lesion: a 6 × 7 mm brown macule with irregular borders on a background of speckled pigmentation. (**C**) Dermoscopy reveals an atypical reticular pigment network, areas with out-of-focus pigmentation, and discrete irregular radial lines at the periphery. (**D**) Histopathology (H&E, 20×) shows a lentiginous proliferation of atypical melanocytes with pagetoid upward migration, overlying a dermal benign melanocytic nevus. (**E**) Immunohistochemical staining with HMB45 (10×) highlights intraepidermal melanocytic proliferation with pagetoid spread and upward extension, supporting a diagnosis of superficial spreading melanoma.

**Figure 3:**
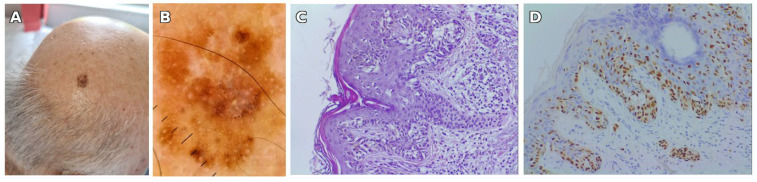
**Third melanoma (2022) on the scalp, outside the SLN.** A third melanoma, superficial spreading type, found at screening on the scalp, at a distance from the SLN. (**A**) Clinical image of the scalp showing a pigmented, irregular, and palpable lesion (14 × 10 mm) in an elderly patient, located away from the sentinel lymph node site. (**B**) Dermoscopic evaluation reveals an atypical pigment network with a blue-gray cloud, eccentric structureless zones, and pink to gray areas suggestive of malignancy. (**C**) Histopathologic section (H&E, 20×) demonstrating pagetoid spread of atypical melanocytes and invasion into the papillary dermis, characteristic of superficial spreading melanoma. (**D**) Immunohistochemistry with SOX10 (20×) highlights the melanocytic proliferation invading the papillary dermis (Clark level II, Breslow thickness 0.4 mm), without evidence of ulceration.

Nine years later, in 2022, the patient was seen in our clinic again for a changing spot within the SLN, this time on the left arm. Among the few darker spots within the SLN, the changing one was a dark brown macule 6 × 7 mm, on dermoscopy showing an atypical reticular network with out-of-focus, light brown areas and discrete, irregular peripheral radiating lines (Figure 2). At the whole body screening, a pigmented lesion stood out on the posterior parietal scalp, unnoticed by the patient. It was a thin, brown-black plaque, irregular, 14 × 10 mm. Dermoscopy showed an irregular reticular network, one eccentric blue-gray cloud, and a structureless area of pink and gray on more than 10% of the surface (Figure 3).

Both lesions were excised with 5 mm margins. The pathological report showed on the left arm superficial spreading melanoma in situ developed on a compound nevus with moderate to severe dysplasia; on the parietal scalp invasive superficial spreading melanoma, Clark II without ulceration, Breslow index 0.3 mm (pT1a). Both were confirmed by immunohistochemistry (Figure 2 and Figure 3); for the invasive melanoma of the scalp, the proliferation index Ki-67 exceeded 5% in the dermal component. Both lesions were re-excised: the one on the arm in our department and the one on the scalp in the surgery department. After 2 years and 6 months, he had no recurrence, and then he was lost to follow-up. Nine years later, in 2022, a second melanoma developed on the left arm, again within the SLN (Figure 2).

During the same visit in 2022, a third melanoma was detected on the parietal scalp, outside the SLN (Figure 3).

The sequence of melanoma events in this patient, together with their clinical and pathological features, is summarized in Table 1. The sequence of melanoma events in this patient is summarized in Table 1, providing a structured overview of the clinical course and forming the basis for the discussion that follows.

This individual case illustrates the clinical and histopathological features of melanoma arising in SLN and provides a natural transition into the broader synthesis of published cases presented below.

### 3.2. Systematic Review Findings

**Figure 4:**
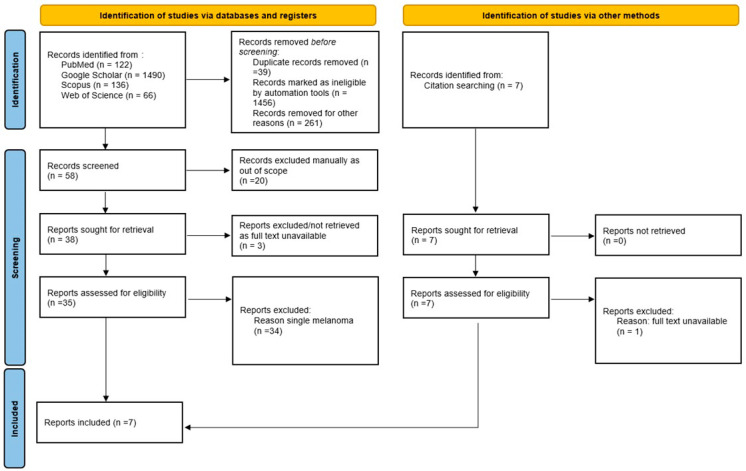
PRISMA 2020 flow diagram illustrating the selection process of studies included in the systematic review of multiple melanomas on speckled lentiginous nevus (SLN). As the available evidence consisted almost exclusively of case reports and small series, the synthesis was qualitative. A total of seven published studies were included in the qualitative synthesis. In addition, we report one illustrative case from our institution; together, these eight sources describe 52 patients with melanoma on SLN, of whom 9 (17.3%) had multiple melanomas (24 tumors). Among the 52 patients, nine (17.30%) developed multiple primary melanomas, accounting for a total of 24 individual tumors, and in 43 patients, melanoma developed as a single lesion. Only these nine cases of multiple melanomas were included in the synthesis presented in this section. Each patient had between two and four melanomas. Their clinical and histopathological characteristics are summarized in Table 2, while the study selection process is illustrated in Figure 5.

A total of 41 eligible full-text articles published between 1957 and 2025 were identified through a comprehensive systematic search across PubMed, Scopus, Web of Science, and Google Scholar. Of these, 35 articles were retrieved through direct database queries and six via citation searches. The included studies described 51 patients with melanoma arising within speckled lentiginous nevus (SLN). Including our illustrative case, the final cohort consisted of 52 patients. The study selection process is detailed in the PRISMA 2020 flow diagram (Figure 4).

**Table 2:** Clinical and histopathological details of the patients with multiple melanomas on SLN found in the literature.

Number	Author, Year	Patient		Features of Nevus Spilus		Features of Malignant Melanoma				
		Sex	Age at Melanoma Diagnosis	Size of SLN	Location of SLN	Number of Melanomas	On/at Distance from SLN	Synchronous/Metachronous	Type of Melanoma	Breslow (mm)
1	Abecassis, 2006 [24]	M	32	Medium (10 cm)	Intergluteal fold	2	On SLN	Synchronous	Nodular	3.5 mm
			32				On SLN	Synchronous	SSM	1.25 mm
2	Boot-Bloemen, 2017 [30]	M	35	Large (over 40 cm)	Trunk, arm	3	At distance	Synchronous	Unspecified	3.6 mm
			41				On SLN	Metachronous	SSM	0.65 mm
			41				On SLN	Metachronous	Unspecified	0.62 mm
3	Brito, 2017 [29]	M	83	Large (27 × 10 cm)	Arm, forearm	4	On SLN	Synchronous	SSM	2.51 mm
			83				On SLN	Synchronous	SSM	1.18 mm
			83				On SLN	Synchronous	Unspecified	In situ
			83				At distance	Synchronous	Unspecified	In situ
4	Ly, 2011 [31]	F	47	Large (over 20 cm)	Lower limb and loin	2	On SLN	Synchronous	SSM	0.75 mm
			51				On SLN	Metachronous	SSM	0.70 mm
5	Ly, 2011 [31]	F	49	Large (over 20 cm)	Arm	3	On SLN	Synchronous	SSM	In situ
			49				On SLN	Synchronous	SSM	In situ
			52				On SLN	Metachronous	SSM	In situ
6	Meguerditchian, 2009 [28]	M	60	Medium (15 × 10 cm)	Leg (calf)	2	On SLN	Synchronous	Unspecified	0.8 mm
			60				On SLN	Synchronous	Unspecified	0.95 mm
7	Piana, 2006 [27]	M	28	Medium (5 cm)	Arm	3	On SLN	Synchronous	SSM	1 mm
			28				On SLN	Synchronous	SSM	0.7 mm
			28				On SLN	Synchronous	SSM	0.3 mm
8	Sansoni, 2024 [18]	M	72	Medium (5.5 × 1.8 cm)	Trunk	2	On SLN	Synchronous	SSM	0.3 mm
			72				On SLN	Synchronous	Unspecified	In situ
9	Our case	M	66	Large (over 40 cm)	Trunk, arm	3	On SLN	Synchronous	SSM	In situ
			76				On SLN	Metachronous	SSM	In situ
			76				At distance	Metachronous	SSM	0.3 mm

Clinical and histopathological details of the nine patients with multiple melanomas on SLN identified in the literature and in our illustrative case. These represent 9 of 52 total patients with melanoma on SLN (17.3%). Abbreviations: SSM—superficial spreading melanoma; SLN—speckled lentiginous nevus.

**Figure 5:**
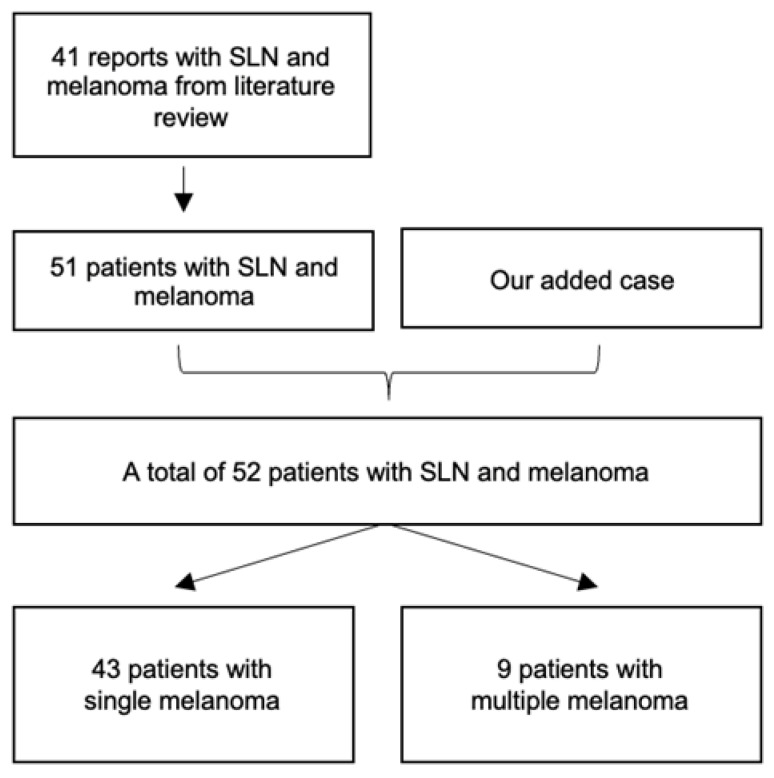
Screening process and results.

Of the nine patients, seven were male and two were female, indicating a male predominance. The mean age at diagnosis of the first melanoma was 52.4 years (standard deviation: 19.1 years; range: 28–83 years). All patients had either medium-sized (5–19.9 cm) or large (≥20 cm) SLNs. No melanomas were reported in association with small SLNs. The affected SLNs were most commonly located on the trunk and upper limbs.

In total, 21 of the 24 melanomas (87.5%) developed within the boundaries of the SLN, while three arose at distant sites—two on the back (in patients with SLNs on the arm or trunk) and one on the scalp. Nineteen melanomas (79.2%) were diagnosed synchronously, while five (20.8%) were metachronous, with a time interval between diagnoses ranging from three to ten years. Of the synchronous melanomas, 17 developed within the SLN and two at distant cutaneous sites. Among the metachronous lesions, four were within the SLN and one occurred remotely.

Histopathological data were available for 17 of the 24 tumors. The majority were superficial spreading melanoma (16 of 17; 94.1%), and only one was classified as nodular melanoma. In cases of metachronous presentation, the subsequent melanomas exhibited the same histologic subtype as the initial tumor in two-thirds of cases (66.7%).

Breslow thickness was reported for all tumors. Eight lesions (33.3%) were in situ, for which thickness is not applicable. Among the 16 invasive melanomas, the mean Breslow thickness was 1.17 mm (median 0.95 mm, IQR 0.56–1.40 mm), with values ranging from 0.3 mm to 3.6 mm. All calculations were performed using invasive tumors only (*n* = 16). Primary invasive melanomas had a higher average Breslow thickness (1.40 mm) compared to subsequent invasive melanomas (0.56 mm), reflecting earlier detection upon surveillance.

Follow-up information was available for five of the nine patients. All five remained free of distant metastases over periods ranging from 10 to 30 months. No melanoma-related deaths were reported during follow-up.

## 4. Discussion

This systematic review consolidates available evidence on multiple melanomas developing in SLN, providing a comprehensive synthesis of case-level data extracted from 41 publications, complemented by an illustrative new case. Our findings underscore distinct clinical patterns and potential risk factors within this rare subset.

The chronology of melanoma development in our illustrative patient (Table 1) highlights several important clinical lessons. First, the 9-year interval between the first and second melanomas underscores the need for long-term surveillance in patients with large SLN, as new lesions may arise after many years of apparent stability. Second, the synchronous occurrence of the second and third melanomas in 2022 illustrates the potential for multiple independent malignant events to develop simultaneously, both within and outside the nevus. Finally, the detection of scalp melanoma during full-body screening emphasizes the importance of examining not only the SLN itself but also the entire skin surface, as high-risk patients may also develop melanomas at distant sites.

These findings highlight the need for rigorous, long-term dermatologic monitoring in patients with large or segmental SLN, particularly after the first melanoma diagnosis, given the risk of synchronous and metachronous lesions.

Speckled lentiginous nevus, also known as nevus spilus, is a patch of light brown pigmentation containing a variable number of darkly pigmented macules and papules [33]. The term “speckled lentiginous nevus” was first proposed by Altman and Banse in 1992. Other terms that have been used are speckled nevus [34], nevus on nevus [4,35], and spotty nevus [3]. This inconsistent terminology reflects the rarity of this condition and the process of understanding its characteristics. When it appears, the light brown macule is discrete, and the lesion becomes evident after months or years when the pigmented macules and papules develop. Thus, the term speckled lentiginous nevus (SLN) better characterizes the biological nature of the lesion, as compared to the term nevus spilus (*spilus* meaning spot in Greek, not “spotty” or “spotted”). Based on its natural history, SLN is considered a variant of congenital melanocytic nevus.

The background tan macule presents histologically increased melanin within basal keratinocytes (like a café-au-lait spot) or an increased number of melanocytes (like lentigo simplex) [9]. It differs from the classic café au lait spot by the presence of melanosome macroglobules that result from aberrant melanogenesis and autophagic processes, with consequent melanin autosome formation [36,37]. The “spots” in SLN have a wide spectrum of clinical and histological appearances, ranging from junctional or compound nevi to, less frequently, lentigines, intradermal, blue, or Spitz nevi [12,19,20]. Hairy SLN may also exist [2,38].

The main differential diagnosis of SLN is an agminated melanocytic nevus, where the background tan macule is missing [38,39]. Another differential diagnosis is with NS-like CMN (nevus spilus-like congenital melanocytic nevus), which shares a tan background with classic SLN but differs phenotypically and genetically. In NS-like CMN, the superimposed lesions are typically larger and darker congenital melanocytic nevi, often with hypertrichosis, and the somatic mutation is most frequently in the HRAS gene [40]. By contrast, classic SLN is more often associated with NRAS or BRAF mutations [6,41,42]. Because of the higher melanoma risk in congenital melanocytic nevi compared with SLN, we excluded cases of NS-like CMN from our analysis [40].

The malignant potential of SLN became evident, especially during the last 25 years when most of the cases of melanoma, single and multiple [18,24,27,28,29,30,31], on SLN have been published.

Our study is the largest systematic review of patients with SLN reported to develop melanoma compared to the existing literature [18,24,31].

The presence of multiple melanomas contiguous with SLN and at long distances might suggest the role of SLN, like any type of melanocytic nevus, as both precursor and marker for melanoma development, alongside other common risk factors.

The mean age when the first melanoma appeared in patients with SLN in our review was not different from the age of patients with SLN who developed a single melanoma (mean age 49–55 years) [2,9]. This age range is also comparable to that of patients with melanomas arising in small and medium-sized congenital melanocytic nevus [43], but significantly older than the early-onset melanomas (childhood and adolescence) observed in giant CMN [44]. These findings suggest that SLN may follow a prolonged latency period before malignant transformation, likely caused by cumulative environmental exposure or gradual acquired somatic mutations over time. In contrast, the median age in patients with multiple primary melanomas, irrespective of the presence of NS, is reported to be around 71 years [45], indicating a different timeline dynamic.

Most patients with multiple melanomas on SLN in this review are men (7:2), while published studies for single and/or multiple melanomas on SLN show women are most frequently affected [46]. While the limited number of cases constrains interpretation, this finding suggests potential sex-related differences in the biology, clinical behavior, or surveillance patterns of SLN. Behavioral factors such as delayed medical visits in men or differences in self-examination and skin monitoring may contribute to this pattern [47]. Hormonal or genetic factors could also play a role in modulating the risk of malignant transformation in SLN over time, but further cases and RCTs are needed to conclude.

Among patients with SLN and melanoma, there is a rate of 16.7% (9/52) of patients who develop multiple melanomas. Although the reported cases are relatively few, the rate is significantly high and comparable with the highest reported rate of 18.1% in the general MPM population [48], suggesting an increased predisposition for multiple melanomas in this subgroup. This is particularly relevant given that rates reported in many other large studies concerning multiple melanomas in the general population range from 3.7% to 9% [45,47]. The minimum time interval between diagnoses of the primary and subsequent primary melanomas in patients with SLN was 3 years, while in studies not focusing on patients with SLN, subsequent primary melanomas were diagnosed in 52% of cases in the first year after the first melanoma [45,49,50].

This review shows that multiple melanomas (almost 80%) SLN-related are predominantly synchronous, and 20% are subsequent, at a maximum time interval of 10 years. This pattern may differ from the general population with multiple primary melanoma (MPM), in which metachronous melanomas seem to be more frequent. For example, in the classic cohort described by Slingluff et al., 64% of secondary melanomas were metachronous and only 36% were synchronous [51]. Similarly, Kelly et al. reported that among 62 patients with MPM, 83% of second melanomas were metachronous, with a mean time interval of 5.4 years, and only 18% were diagnosed synchronously [52]. The multiple independent malignant events within the area of medium/large SLN suggest a field cancerization effect in these unstable types of nevi. Increased clinical alertness and focused examination of SLN areas are needed once a first melanoma has been diagnosed.

Recent melanomas may present with subtle clinical features, which may cause them to be overlooked during routine clinical examination of an SLN, where multiple pigmented macules and papules coexist. In our case, at least three dark brown maculopapules looked similarly suspicious at clinical examination of SLN with the naked eye in terms of color and size. Dermoscopic evaluation revealed atypical features in only one of them (located on the left arm), which prompted its excision and histopathological confirmation as melanoma. Therefore, dermoscopy plays an essential role in the identification of early atypical features and differentiation of clinically similar lesions correlated with the history of change of the lesion.

Superficial spreading melanoma is the most frequent (94%) histologic type of SLN-related melanoma in this study. This finding is consistent with data from the general melanoma population, where SSM represents the predominant histopathologic type. For example, a study from northwestern Romania reported SSM in 74.0% of men and 80.8% of women with melanoma [53], while larger epidemiologic data cite an incidence of 59.5% [54]. Multiple SSMs in these patients appear as focal darkening within the speckled area or develop irregular margins, allowing early identification by comparison with the other speckles with the help of dermoscopy. The radial growth phase typical of SSM is associated with a better prognosis, as it allows for earlier detection when the tumor is still thin, which is reflected in our data by a relatively low mean Breslow thickness of 1.17 mm. The multifocal character of synchronous melanoma within the SLN underlines the importance of videodermoscopic follow-up of these nevi and total body mapping as well, as distant synchronous or metachronous melanoma may occur.

Studies of multiple cutaneous melanomas in patients not specified as having SLN showed a tendency for thinner subsequent melanoma [47], which was also found in this study. This is probably due to increased clinical vigilance and early detection following a primary diagnosis. Our patient was an exception to this tendency, as one of the subsequent melanomas located on the posterior parietal scalp, a hard-to-reach area of the body, was thicker than the primary melanoma and was detected during the whole-body screening. This case shows how certain anatomical locations, such as the scalp, can lead to delayed diagnosis of melanoma. Therefore, full body examination at each visit during long-term follow-up in patients with SLN and melanoma is recommended for early detection of new lesions. Doctors should also educate patients with SLN on self-skin examination to become aware that the „spots” scattered on a tan background may change in color, size, and shape.

In our series, all melanomas appeared on SLN ≥ 5 cm (medium, large, and giant), with a little more than half on large and giant types. Abecassis et al. [24] observe that melanoma appears most frequently (10/13 cases) on SLN ≥ 4 cm, suggesting a higher risk for melanoma in this category of SLN. Similarly, Boot-Bloemen et al. found a 60% risk of melanoma in segmental nevi >20 cm, with an even higher risk noted in lesions >40 cm [30]. Corradin et al. found that 60% of melanomas appear on small and medium NS (medium size 7.4 cm), and 40% on zosteriform and giant NS [9]. The increasing size is also a risk factor in congenital nevi [9,30]. The risk seems to be higher in large SLN lesions compared to congenital pigmented nevi of the same size, but it is similar in SLN and CMN of small and medium sizes [9,30].

Two primary types of SLN have been described, depending on the spotting within the lesion [5,29]: macular and papular. In macular SLN, the darker speckles are flat, and they correspond histopathologically to nests of melanocytes at the dermo-epidermal junction at the tips of dermal papillae. The pattern always follows a checkerboard-like model, though this may not be obvious in small lesions. Studies suggest that the lesion is usually stable rather than growing or changing over time [5]. In the papular variant, along dark macules, there are papules and nodules unevenly distributed, corresponding histopathologically to dermal or compound nevi [5].

The relationship between SLN type and melanoma risk remains controversial. Some authors suggested that both the incidence and the risk of developing melanoma are higher in the macular form [8,9], while others show that melanoma develops more frequently on the papular form of SLN [9].

The prevalence of SLN was not found to be significantly different in 105 adults with melanoma (4.8%) versus 601 controls (2.3%) [7]. Another study found an incidence of 1% for NS (27 cases), none with malignant changes, in 2134 patients for melanoma follow-up [55]. Among 946 patients with a diagnosis of NS, only 2 cases with melanoma were found [56].

The major risk factors for developing melanoma are the presence of SLNs very early in life (the congenital type), a size of more than 4 cm, and giant or zosteriform forms [18]. Excessive UV exposure in genetically predisposed patients is also considered a trigger for multiple melanomas, based on the presence of multiple lentigines observed clinically within SLN and solar damage found in histopathology in these lesions [31].

### 4.1. Patient Risk Profile

Our synthesis suggests that the patients at highest risk for melanoma development within SLN are those with large or segmental lesions measuring at least 5 cm, particularly when distributed in a zosteriform pattern or with irregular morphology such as heterogeneous pigmentation or papular components. Male sex appeared to predominate among reported cases with multiple melanomas, and fair Fitzpatrick skin phototypes (I–II) may further increase susceptibility due to reduced natural photoprotection. Additional contributing factors include a positive personal or family history of melanoma or non-melanoma skin cancers and high cumulative ultraviolet exposure, particularly intermittent intense sun exposure or a history of sunburns. The coexistence of these clinical, phenotypic, and epidemiologic features may delineate a subgroup of SLN patients who warrant the closest dermatological and dermoscopic surveillance.

Recognizing this high-risk patient profile provides a rationale for tailoring surveillance strategies, which should be risk-adapted and proactive in order to facilitate early detection and timely intervention.

### 4.2. Clinical Implications and Follow-Up Strategies

The recognition of a high-risk patient profile provides a foundation for translating these findings into clinical practice. We recommend full-body skin examinations every 6–12 months for patients with medium-to-large or segmental SLN. In patients with a history of melanoma, closer intervals of every 3–6 months are advisable in the years immediately following the first diagnosis, with subsequent adjustment according to individual risk factors [57]. Baseline and sequential dermoscopic imaging, ideally supported by digital videodermoscopy, can aid in detecting subtle changes within speckles that may indicate early malignant transformation. Where available, reflectance confocal microscopy (RCM) may serve as a valuable adjunct for evaluating evolving or atypical speckles. Patient education is equally important, empowering individuals to perform regular self-monitoring and to promptly report new or changing pigmented lesions. These surveillance recommendations are preliminary and must be adapted to each patient, given the limited evidence base and the descriptive nature of currently available data.

Other relevant risk factors involved are light Fitzpatrick skin type (I or II) and a positive personal history of non-melanoma skin cancers [58]. 

The malignant potential of SLN remains unclear, with reported risk estimates ranging from 0.13% to 0.2% [24,29]. These values are largely based on case series and literature reviews and may underestimate the true risk due to underreporting and limited long-term surveillance data. Interestingly, these estimates suggest that the risk of melanoma occurrence in SLN may exceed that reported for giant congenital hairy nevi, which have long been considered among the pigmentary lesions with the highest risk of malignant transformation [59].

After a diagnosis of melanoma in an SLN, the excision of the entire lesion should be considered, if feasible, but prophylactic excision of the SLN is not encouraged. For large or segmental SLN, periodic follow-up through dermoscopy and sequential dermoscopic imaging, corroborated with reflectance confocal microscopy (RCM) [60,61], if available, assists in early diagnostic melanoma. Melanoma that arises within the SLN is approached similarly to any other melanoma.

Multiple SLN-related melanomas have an excellent prognosis when diagnosed early. In our review, patients with the longest follow-up periods (ranging from 10 months to more than 2 years) remained disease-free, including our patient who was found to be well 2 years and 6 months after diagnosis. Synchronous melanomas do not confer a worse prognosis in SLN patients compared to the general population, in which Xiong et al. reported that synchronous multiple primary melanomas are associated with worse overall survival and are a significant risk factor for poorer outcomes [62].

Our study is the largest systematic review of patients with SLN reported to develop melanoma compared to the existing literature [18,31]. Its strength lies in the high number of patients with SLN who develop melanoma and in the fact that it is the only systematic review assessing multiple melanomas in patients with SLN.

### 4.3. Limitations

This study has several limitations. Although designed and conducted as a systematic review in accordance with PRISMA guidelines, the available evidence consisted almost exclusively of case reports and small case series. This limited and heterogeneous dataset precluded meta-analysis and the application of a robust risk-of-bias assessment. Selection bias, publication bias, and the underreporting of negative or asymptomatic cases may further skew the available evidence. Moreover, clinical and histopathological details were not uniformly reported across cases, and long-term follow-up was often lacking. Consequently, our findings should be interpreted as descriptive and exploratory, providing a qualitative mapping of the literature rather than definitive risk estimates.

Although this work was designed and conducted as a systematic review following PRISMA guidelines, the evidence base consisted almost exclusively of case reports and small series. This limited and heterogeneous dataset precluded quantitative pooling of results and the application of a robust risk of bias assessment. Consequently, the findings are descriptive in nature and should be interpreted as a qualitative synthesis. Although a complete search of major databases was carried out, there is still a strong possibility that cases in less accessible languages or unindexed journals might have been omitted.

The small number of reported cases makes it difficult to accurately assess the risk of developing melanoma in patients with SLN. In most of the reports analyzed, there is a lack of long-term data on the possibility of melanoma occurring after the follow-up period. There was insufficient information about the macular, papular, or dysplastic nature of SLN to use these parameters in our study. This review emphasizes that speckled lentiginous nevi (SLN), especially large and segmental types, carry a clinically relevant risk for the development of multiple melanomas. The high rate of synchronous lesions within SLN, often indistinguishable from benign components on visual inspection alone, underscores the importance of structured dermoscopic monitoring and total-body skin examinations. Incorporating SLN into melanoma risk assessment protocols—particularly when associated with large lesions or a history of melanoma—may lead to earlier detection of secondary melanomas and improved patient outcomes. Our findings support a shift in clinical practice toward long-term, proactive surveillance of patients with SLN rather than viewing these lesions as uniformly low-risk. Given that many included reports lacked standardized follow-up and may suffer from publication bias, these findings may underestimate the true risk. As this review primarily includes case reports and small series, the synthesis must be regarded as descriptive and exploratory. The evidence base is inherently heterogeneous, retrospective, and subject to publication bias, which may skew the available data toward more severe or unusual presentations. Consequently, our findings should be interpreted with caution and considered as a qualitative mapping of the literature rather than a quantitative estimate of risk.

### 4.4. Future Directions

Future research should aim to strengthen the evidence base on melanoma risk in patients with speckled lentiginous nevus. Multicenter registries and collaborative international databases would allow for the prospective collection of standardized clinical, dermoscopic, and histopathological data [63]. Such efforts could provide more precise risk stratification, clarify the influence of patient and lesion characteristics, and inform the development of evidence-based guidelines for surveillance. Additionally, prospective longitudinal studies are needed to assess the natural history of SLN and to evaluate the effectiveness of risk-adapted [64] follow-up strategies in improving early detection and outcomes.

Further prospective studies are needed to understand the biological behavior of SLN and its role in melanoma predisposition. Until then, clinical vigilance, regular dermoscopic monitoring, and patient education remain essential in managing SLN patients, particularly those with large or congenital lesions.

### 4.5. Key Clinical Message

Patients with large or segmental speckled lentiginous nevi (SLN) are at increased risk of developing multiple melanomas, often synchronously. Regular, risk-adapted surveillance combining full-body examination, dermoscopic monitoring, and patient self-awareness is essential for early detection and improved outcomes.

## 5. Conclusions

This systematic review demonstrates that large or segmental SLNs may carry a greater risk of multiple melanomas than previously appreciated. Most lesions are of the superficial spreading subtype and frequently occur synchronously. Dermatologists should conduct regular dermoscopic and full-body skin examinations in SLN patients, especially those with medium and large lesions, to enable early detection and intervention. Future multicenter registries and collaborative international studies are recommended to collect prospective data and clarify melanoma risk stratification in patients with SLN. Given the reliance on case reports and small series, these conclusions are necessarily descriptive and exploratory; they provide a qualitative synthesis of the available evidence rather than definitive risk estimates.

## Figures and Tables

**Table 1:** Chronology and clinicopathological characteristics of the three melanomas in the illustrative patient with speckled lentiginous nevus (SLN).

Year (Age)	Site	Relation to SLN	Clinical Features	Histological Type	Breslow (mm)	Stage
2013 (66)	Left shoulder	Within SLN	Verrucous black plaque, 10 × 8 mm, eccentric pigmentation	Superficial spreading melanoma, in situ	–	pTis
2022 (75)	Left arm	Within SLN	Flat brown macule, 6 × 7 mm, atypical reticular network on dermoscopy	Superficial spreading melanoma, in situ	–	pTis
2022 (75)	Posterior parietal scalp	Outside SLN	Irregular brown-black plaque, 14 × 10 mm	Superficial spreading melanoma, invasive (Clark II)	0.3	pT1a

Chronology and clinicopathological features of the three melanomas diagnosed in the illustrative patient with speckled lentiginous nevus. The first two melanomas developed within the nevus (in situ), while the third arose outside the nevus (invasive). This sequence illustrates both metachronous and synchronous development of melanoma.

## Data Availability

The data supporting the findings of this study are available from the corresponding author upon reasonable request. Due to the nature of case report data and patient confidentiality agreements, some clinical details may be restricted. No publicly archived datasets were generated or analyzed during the current study. Note on Methodology: This study was designed and conducted as a systematic review in accordance with PRISMA guidelines. However, because the available evidence consisted almost exclusively of case reports and small series, quantitative pooling and formal risk-of-bias assessment were not feasible. The synthesis is therefore qualitative, descriptive, and exploratory in nature, aiming to map and contextualize the fragmented literature on multiple melanomas arising in speckled lentiginous nevus.

## Abbreviations

The following abbreviations are used in this manuscript:

AJCC	American Joint Committee on Cancer
CMN	Congenital Melanocytic Nevus
HE	Hematoxylin and Eosin
HMB45	Human Melanoma Black 45 (Immunohistochemical Marker)
IRB	Institutional Review Board
Ki-67	Proliferation Marker Ki-67
MPM	Multiple Primary Melanoma
NS	Nevus Spilus
pT1a	Pathologic Tumor Stage 1a
PRISMA	Preferred Reporting Items for Systematic Reviews and Meta-Analyses
RCM	Reflectance Confocal Microscopy
RCT	Randomized Controlled Trial
SD	Standard Deviation
SLN	Speckled Lentiginous Nevus
SOX10	SRY-Related HMG-box 10 (Immunohistochemical Marker)
SSM	Superficial Spreading Melanoma
UV	Ultraviolet
